# The Interplay of Anxiety, Depression, Sleep Quality, and Socioeconomic Factors in Somali Hemodialysis Patients

**DOI:** 10.3390/brainsci14020144

**Published:** 2024-01-30

**Authors:** Samet Kose, Nur Adam Mohamed

**Affiliations:** 1 Department of Psychiatry and Behavioral Sciences, Mogadishu Somalia Turkey Recep Tayyip Erdogan Research and Training Hospital, Mogadishu, Somalia; nurmohed01@gmail.com; 2 Department of Psychiatry, Basaksehir Cam Sakura City Hospital, Health Sciences University, Istanbul 34480, Türkiye

**Keywords:** anxiety, depression, hemodialysis, kidney failure, quality of sleep, socioeconomic factors, Somalia

## Abstract

Objective: This study aimed to assess anxiety, depression, and sleep quality in kidney failure patients receiving hemodialysis (HD) in Somalia and examine the relationship between anxiety, depression, and sleep quality. Methods: We conducted a study with 200 kidney failure patients on HD treatment for over 3 months. Participants completed sociodemographic questionnaires, the Patient Health Questionnaire-9 (PHQ-9), the Hospital Anxiety and Depression Scale (HADS), the Insomnia Severity Index (ISI), and the Pittsburgh Sleep Quality Index (PSQI). Results: Among the 200 participants (mean age = 52.3; SD = 14.13), 58.5% were men, 64% had CKD for 1–5 years, and 52.6% received HD for 1–5 years. Depressive symptoms were found in 61.5% (PHQ-9) and 37.5% (HADS depression subscale) of HD patients. Poor sleep quality (PSQI) was observed in 31.5% and significantly correlated with PHQ-9 (*r_s_* = 0.633), HADS anxiety (*r_s_* = 0.491), and HADS depression (*r_s_* = 0.529). The ISI score correlated significantly with PHQ-9 (*r_s_* = 0.611), HADS anxiety (*r_s_* = 0.494), and HADS depression (*r_s_* = 0.586). All PSQI components correlated with depression and anxiety, except sleep medication use. Hierarchical regression analysis revealed that HADS anxiety (*β* = 0.342) and HADS depression (*β* = 0.372) predicted ISI scores. HADS anxiety (*β* = 0.307) and HADS depression (*β* = 0.419) predicted PSQI scores. Conclusions: Higher anxiety and depression levels negatively correlated with various dimensions of sleep quality in kidney failure patients. Early identification and appropriate management of these psychological disturbances are crucial for enhancing patients’ overall quality of life.

## 1. Introduction

Chronic kidney disease (CKD) is a prevalent non-communicable illness that poses a growing global health concern [1]. It is estimated that 11–13% of the global population have CKD, with stage 3 representing the most prevalent form [2]. According to the National Kidney Foundation, CKD ranks as the 8th leading cause of death in the United States [3]. Numerous studies have demonstrated a close correlation between CKD and an array of social and psychological challenges, such as depression, anxiety, and a diminished quality of life [1,4]. The psychological well-being of individuals with CKD is influenced by various factors, including the burden associated with the condition, ongoing medical care, coexisting chronic illnesses, alterations in self-perception, fear of mortality, dietary and functional limitations, and the substantial cost of care [1,4].

Anxiety and depression are psychiatric disorders of significant prevalence among patients undergoing HD as a result of CKD, and they frequently remain undiagnosed and untreated [1,5]. These disorders can detrimentally impact functional capacity, contribute to suicidal ideation, compromise the immune system, impair nutritional status, and disrupt sleep quality, ultimately leading to heightened morbidity and mortality in this patient population [6]. Numerous studies have highlighted significant variation in the prevalence of anxiety and depression among individuals with kidney failure across different regions, including Kenya, Egypt, Sudan, Ethiopia, Iran, and Europe. In Kenya, a recent study reported anxiety and depression rates of 45% and 72.5%, respectively, among HD patients [7]. Meanwhile, in Egypt, rates of anxiety and depression were found to be 33.7% and 31.9% in HD patients [8]. In Sudan, depression prevalence among HD patients was reported at 68% [9], and in Ethiopia, 60.3% of HD patients were diagnosed with depression [10]. Iran’s study showed that approximately 45% of HD patients experienced depression [11]. Similarly, in a Dutch observational prospective cohort study, 22% of patients experienced anxiety and 42% reported symptoms of depression [12]. Additionally, in Latin America, a longitudinal study in Brazil reported anxiety rates of 27.9% and depression rates of 31.2% among HD patients [13]. In a sample from Singapore, a study revealed anxiety and depression rates of 31.8% and 39.6%, respectively, among HD patients [14]. These consistent findings over time and across different regions underscore the significance of mental health challenges faced by individuals with kidney failure undergoing HD.

Moreover, patients with kidney failure undergoing regular HD frequently encounter sleep disorders [15,16]. The prevalence of sleep disturbances among these individuals has been reported to range from 40% to 80%, significantly surpassing rates observed in the general population [17,18]. Kidney failure patients may experience various sleep disorders, including restless leg syndrome (RLS), obstructive sleep apnea (OSA), central disorders of hypersomnolence, insomnia, sleep-related breathing disorders, excessive daytime sleepiness, sleep–wake disorders, nightmares, narcolepsy, night-time waking, sleepwalking, and parasomnias [19,20].

Sleep quality is multifaceted in nature, encompassing factors like sleep efficiency, latency, wake after sleep onset, and individuals’ perception of sleep quality and level of sleepiness. Across multiple studies, a consistent pattern emerges: a high prevalence of poor sleep quality among patients with kidney failure. For instance, Kamal et al. reported that 62.2% of HD patients in Egypt experienced poor sleep quality [21]. Similarly, a study conducted in Kenya revealed that 69.6% of HD patients reported poor sleep quality [22]. In Senegal, Tondi et al. discovered that 88% of HD patients suffered from sleep disturbances, with insomnia and obstructive sleep apnea being the most prevalent [23]. Furthermore, Mujahid et al. found that 66% of their study participants had poor sleep quality [16]. These collective findings underscore the substantial burden of sleep disturbances and poor sleep quality among individuals undergoing HD treatment for kidney failure. They emphasize the pressing need for further research and interventions to address sleep-related issues in this patient population.

In considering the emotional parameters of patients, it is essential to acknowledge the potential influence of cultural contexts on attitudes. As discussed by Giannouli and Syrmos [24], attitudes towards health issues, including kidney diseases, can vary significantly across different cultures. Understanding these cultural nuances becomes pivotal in enhancing our comprehension of emotional responses among patients, ultimately contributing to more effective healthcare strategies and interventions. In the context of Somalia, where healthcare disparities and limited access to specialized services are prevalent, it is crucial to understand the determinants of sleep health. While previous research has examined the impact of anxiety and depression on sleep quality among HD patients in Somalia, there is a lack of studies exploring the broader socioeconomic factors contributing to sleep health disparities in this population. A recent systematic review conducted in Iran and Saudi Arabia [25] found no significant associations between socioeconomic determinants and sleep components among adult populations. Simonelli et al.’s study [26] revealed higher prevalence of short sleep duration and insomnia in unsafe, noisy neighborhoods among US Hispanic/Latino individuals. Adverse neighborhood factors are known risk factors for negative health outcomes, with sleep disturbances potentially mediating their impact. Our study on anxiety, depression, and sleep quality among Somali HD patients recognizes the relevance of contextual factors, including neighborhood characteristics, in shaping sleep patterns in this population.

In Africa, there remains a scarcity of research investigating the psychological challenges encountered by individuals with kidney failure. To the best of our knowledge, there is a notable absence of data addressing this topic from Somalia. In our previous work, we have shown significant associations between poor sleep and perceived family support and friends’ support [27]. The present study aims to examine the levels of anxiety, depression, and sleep quality in kidney failure patients undergoing HD treatment within a Somali population. Building upon existing literature highlighting the psychological challenges faced by individuals with kidney failure globally [7,8,9,10,11], our hypotheses are twofold. First, we hypothesize that the prevalence of anxiety and depression will be substantial among Somali HD patients, consistent with findings from diverse cultural contexts. Second, we hypothesize a significant association between anxiety, depression, and sleep quality, aligning with the bidirectional relationship established in previous research [16,21,22,23]. By systematically exploring these hypotheses, our study contributes to a deeper understanding of the psychological well-being of kidney failure patients in Somalia and offers insights for targeted interventions informed by the specific needs of this population.

## 2. Methods

This study used a cross-sectional design and took place at the HD unit of the Mogadishu Somalia Türkiye Recep Tayyip Erdogan Research and Training Hospital in Mogadishu, Somalia. The study included 200 patients (83 women and 117 men) undergoing HD treatment 2–3 times per week. The inclusion criteria were being 18 years of age or older, being on HD treatment for more than three months prior to the study, and being proficient in Somali. The administration of the scales was exclusively conducted in the Somali language. The exclusion criteria were being younger than 18 years of age, being on HD treatment for less than three months prior to the study, having a lack of proficiency in Somali, having a hearing impairment, having a critical illness requiring hospitalization, having a history of depression and anxiety or current use of psychotropic medications, and being female patients who were pregnant. The following assessments were given to all participants: a sociodemographic data form, Patient Health Questionnaire-9 (PHQ-9), Hospital Anxiety and Depression Scale (HADS), Insomnia Severity Index (ISI), and Pittsburgh Sleep Quality Index (PSQI).

### 2.1. Psychometric Scales

Patient Health Questionnaire-9 (PHQ-9). The PHQ-9 is a 9-item questionnaire developed by Kroenke et al. [28] that measures depressive symptoms over the past 2 weeks. Participants rate their experiences on a scale of 0 to 3, where 0 is “not at all” and 3 is “nearly every day”. Scores range from 0 to 27. A score of 5 to 9 is defined as mild depression, a score of 10 to 14 indicates moderate depression, and a score of 15 or higher indicates moderate to severe depression. The Somali version of the PHQ-9 [29] had a Cronbach’s alpha of 0.79. In the current sample, Cronbach’s alpha for the PHQ-9 was 0.76.

Hospital Anxiety and Depression Scale (HADS). The HADS is a 14-item self-report questionnaire developed by Zigmond and Snaith [30] to assess anxiety and depression symptoms in non-psychiatric patients receiving treatment in hospitals and outpatient clinics. It consists of two dimensions: anxiety (HADS-A) and depression (HADS-D), each with 7 items rated on a 4-point Likert scale. Scores of 0–7 are considered normal, 8–10 indicate mild disorder, 11–15 indicate moderate disorder, and 16–21 indicate severe disorder. The Somali version of the HADS [31] had a Cronbach’s alpha of 0.83 for the anxiety subscale and 0.84 for the depression subscale. In the current sample, Cronbach’s alpha was 0.84 for the anxiety subscale and 0.75 for the depression subscale of the HADS.

The Insomnia Severity Index (ISI). The ISI is a 7-item self-reported questionnaire designed by Bastien et al. [32] to evaluate the type, intensity, and impact of insomnia. It assesses the severity of difficulties with sleep onset, maintenance, early morning awakening, sleep dissatisfaction, interference with daytime functioning, noticeability of sleep problems by others, and distress caused by lack of sleep over the past month using a 5-point Likert scale (0 = no difficulty, 4 = very severe problem). The total score ranges from 0 to 28 and categories of insomnia are as follows: no insomnia (0–7), sub-threshold insomnia (8–14), moderate insomnia (15–21), and severe insomnia (22–28). The ISI is available in three forms: patient, clinician, and significant other. The Cronbach’s alpha for the sample was 0.74.

The Pittsburgh Sleep Quality Index (PSQI). The PSQI is a self-assessment questionnaire consisting of 19 self-rated questions and 5 questions rated by a bed partner/roommate [33]. It assesses the individual’s sleep quality over the last 4 weeks. The 19 self-rated questions are added together to create 7 component scores, including subjective sleep quality, sleep latency, sleep duration, sleep efficiency, sleep disruption, sleep drug usage, and daytime dysfunction. Each component is scored on a scale of 0 to 3, with a score of 0 indicating no difficulty and a score of 3 indicating significant difficulty. The total PSQI score, which ranges from 0 to 21, is calculated by combining the seven component scores. A higher total score indicates poorer sleep quality. Patients who obtain a global PSQI score of >5 are referred to as “poor sleepers”, whereas patients who obtain a score of 5 or less are referred to as “good sleepers”. Although bed partner or roommate responses do not count in the overall PSQI total score, they can provide additional insight into the patient’s sleep patterns. The present study’s sample had a Cronbach’s alpha of 0.85 for the PSQI. Deletion of one component would increase Cronbach’s α by 0.02 for Sleep Disturbance and 0.02 for Sleep Medication.

### 2.2. Statistical Analysis

All statistical analysis was conducted using SPSS (Armonk, NY, USA: IBM Corp.), version 26.0. The sample size was calculated using Open Epi version 3.01 by taking alpha 0.05 (95% confidence level), which gave the final sample size of 190 patients [population size (for finite population correction factor or fpc) (*N*): 17,597,511; hypothesized % frequency of outcome factor in the population (*p*): 14.4% ± 5; confidence limits as % of 100 (absolute ± %) (*d*): 5%; design effect (for cluster surveys-*DEFF*): 1]. During data collection, we managed to collect data from 200 patients. Categorical variables were presented as frequencies and percentages, while continuous variables were represented by median (P50), 25th percentile (P25), and 75th percentile (P75). Due to non-normal data distribution, Spearman’s rank-order test was used for correlation analysis.

Hierarchical regression analyses were performed to examine the predictive relationship between anxiety, depression, and sleep quality parameters, which were added to the model in separate steps, with each step building upon the previous one. In the first block, sociodemographics including age, gender, family income level, duration of CKD, and duration of HD were included as control variables, and in the second block, anxiety and depression symptoms were added as the target dependent variables. Hierarchical regression facilitates a more nuanced interpretation of the relationships between variables, as it allows us to evaluate how the inclusion of predictors at each stage changes the model’s fit and the coefficients of the predictors. In our model, age, gender, and family income were entered as categorical variables, and duration of CKD and duration of HD were entered as continuous variables. We employed dummy coding for gender, where the absence of the female category was coded as 0 for males, and the presence of the female category was coded as 1. A *p*-value of less than 0.05 was considered statistically significant.

## 3. Results

This study included 200 participants undergoing HD, with an average age of 52.3 years (SD = 14.13) and an age range of 18 to 86 years. Among the participants, 41.5% were women and 58.5% were men. Most of the participants were married (69.5%) and had an illiteracy rate of 68.5%. The majority had a duration of CKD and HD of 1–5 years (64% and 52.6%, respectively). Hypertension was the reported cause of kidney failure in 58.5% of participants, while diabetes mellitus accounted for 20% of cases (Table 1).

The descriptive characteristics as medians and interquartile ranges, means and standard deviations for the PHQ-9, HADS, ISI, and PSQI dimension scores were displayed in Table 2. Approximately 48% of participants reported mild symptoms of depression (cut-off score of 10) and 13.5% reported moderate-to-severe symptoms of depression according to the PHQ-9 scale (scores of 10–19), while 37.5% reported similar symptoms on the HADS depression subscale (cut-off score of 8). On the HADS anxiety subscale, 7% reported mild to moderate symptoms (cut-off score of 8), with only 0.5% experiencing severe anxiety symptoms. Moreover, 31.5% reported poor sleep quality (total PSQI score >5), and 20.5% had clinically significant insomnia (total ISI score >14) (Table 3).

Age and family income were significantly correlated with the sleep duration component of the PSQI scale (*r_s_* = 0.175, *p* < 0.05; *r_s_* = 0.171, *p* < 0.05, respectively). Gender was significantly correlated with the sleep efficiency component of the PSQI scale (*r_s_* = −0.171, *p* < 0.05). The duration of CKD was significantly correlated with the sleep duration and sleep disturbance components of the PSQI scale, duration of HD, the PHQ-9 scale total score, the HADS anxiety subscale, and the HADS depression subscale (*r_s_* = 0.160, *p* < 0.05; *r_s_* = 0.144, *p* < 0.05; *r_s_* = 0.834, *p* < 0.01; *r_s_* = 0.221, *p* < 0.01; *r_s_* = 0.143, *p* < 0.05; *r_s_* = 0.250, *p* < 0.01, respectively). The duration of HD was significantly correlated with the PSQI scale sleep latency component, the PHQ-9 scale total score, and the HADS depression subscale (*r_s_*= 0.148, *p* 0.05; *r_s_*= 0.168, *p* 0.05; *r_s_*= 0.192, *p* 0.01, respectively). The ISI scale total score was significantly correlated with the PSQI scale total score, the PHQ-9 scale total score, the HADS anxiety subscale, and the HADS depression subscale (*r_s_* = 0.803, *p* < 0.01; *r_s_* = 0.611, *p* < 0.01; *r_s_* = 0.494, *p* < 0.01; *r_s_* = 0.586, *p* < 0.01, respectively). The PSQI scale total score was significantly correlated with the PHQ-9 scale total score, the HADS anxiety subscale, and the HADS depression subscale (*r_s_* = 0.600, *p* < 0.01; *r_s_* = 0.460, *p* < 0.01; *r_s_* = 0.606, *p* < 0.01, respectively). The subjective sleep quality component was significantly correlated with the PHQ-9 scale total score, the HADS anxiety subscale, and the HADS depression subscale (*r_s_* = 0.633, *p* < 0.01; *r_s_* = 0.491, *p* < 0.01; *r_s_* = 0.529, *p* < 0.01, respectively). The sleep latency component was significantly correlated with the PHQ-9 scale total score, the HADS anxiety subscale, and the HADS depression subscale (*r_s_* = 0.453, *p* < 0.01; *r_s_* = 0.419, *p* < 0.01; *r_s_* = 0.502, *p* < 0.01, respectively). The sleep duration component was significantly correlated with the PHQ-9 scale total score, the HADS anxiety subscale, and the HADS depression subscale (*r_s_* = 0.572, *p* < 0.01; *r_s_* = 0.388, *p* < 0.01; *r_s_* = 0.533, *p* < 0.01, respectively). The sleep efficiency component was significantly correlated with the PHQ-9 scale total score, the HADS anxiety subscale, and the HADS depression subscale (*r_s_* = 0.402, *p* < 0.01; *r_s_* = 0.390, *p* < 0.05; *r_s_* = 0.419, *p* < 0.01, respectively). The sleep disturbance component was significantly correlated with the PHQ-9 scale total score, the HADS anxiety subscale, and the HADS depression subscale (*r_s_* = 0.287, *p* < 0.01; *r_s_* = 0.206, *p* < 0.01; *r_s_* = 0.393, *p* < 0.01, respectively). The daytime dysfunction component was significantly correlated with the PHQ-9 scale total score, the HADS anxiety subscale, and the HADS depression subscale (*r_s_* = 0.596, *p* < 0.01; *r_s_* = 0.474, *p* < 0.01; *r_s_* = 0.554, *p* < 0.01, respectively). The PHQ-9 scale total score was significantly correlated with the HADS anxiety subscale, and the HADS depression subscale (*r_s_* = 0.516, *p* < 0.01; *r_s_* = 0.716, *p* < 0.01, respectively). The HADS anxiety subscale was significantly correlated with the HADS depression subscale (*r_s_* = 0.606, *p* < 0.01) (Table 4).

The study found that 44.1% of the variability in the ISI scale total score was accounted for by the inclusion of the PHQ-9 scale total score, the HADS anxiety subscale, and the HADS depression subscale (F(3, 191) = 47.420, *p* = 0.000), resulting in a 41.6% increase in predictive capacity. In hierarchical regression analyses, after adjusting for age, gender, family income level, duration of CKD, and duration of HD in step 2, HADS anxiety symptoms (*β* = 0.342, *p* < 0.01) and HADS depressive symptoms (*β* = 0.372, *p* < 0.01) positively predicted insomnia severity, which explained an additional 40.2% of the variance. The Durbin–Watson statistic showed an acceptable value of 1.844, which falls under the acceptable range of 1.5 to 2.5 and means that no linear autocorrelation was present in the data. Our study revealed that all tolerance values ranged between 0.1 and 1.0, confirming the absence of multicollinearity. Additionally, the VIF value was less than 5, which underscores the absence of multicollinearity in the model. The beta coefficients revealed significant associations between mental health scores and insomnia-related measures. Specifically, a higher HADS anxiety score (beta coefficient of 0.342) was linked to a median increase of 0.3 in the insomnia scores, indicating poor sleep quality. Similarly, a higher HADS depression score (beta coefficient of 0.372) was associated with a median increase of 0.3 in the insomnia scores, indicating poor sleep quality (Table 5a).

Similarly, after adjusting for age, gender, family income level, duration of CKD, and duration of HD in step 2, HADS anxiety symptoms (*β* = 0.307, *p* < 0.01) and HADS depressive symptoms (*β* = 0.419, *p* < 0.01) positively predicted insomnia severity, which explained an additional 39.1% of the variance (Table 5b). The Durbin–Watson statistic showed an acceptable value of 1.745, which falls within the acceptable range of 1.5 to 2.5 and means that no linear autocorrelation was present in the data. Our study revealed that all tolerance values ranged between 0.1 and 1.0, confirming the absence of multicollinearity. Additionally, the VIF value was less than 5, which underscores the absence of multicollinearity in the model. The beta coefficients revealed significant associations between mental health scores and sleep quality measures. A higher HADS anxiety beta coefficient (0.307) was linked to a median increase of 0.3 in the PSQI scores, indicating poor sleep quality. A higher HADS depression beta coefficient (0.419) was associated with a median increase of 0.4 in the PSQI scores, indicating poor sleep quality (Table 5b).

## 4. Discussion

In our study, we examined the relationship between anxiety, depression, and sleep quality among Somali HD patients. Our findings revealed that approximately one-third of HD patients reported mild to moderate depression symptoms (HADS-D), and 7% exhibited similar levels of anxiety (HADS-A). These rates align with prior research, indicating high depression rates in CKD and HD patients, significantly impacting their well-being [34]. Nearly 30% reported poor sleep quality, with one-fifth experiencing clinically significant insomnia, emphasizing the need for sleep hygiene and insomnia management interventions for HD patients [35]. Our analysis established links connecting poor sleep quality (PSQI) and insomnia severity (ISI) with anxiety and depression levels. Additionally, demographic and clinical factors played crucial roles. Positive associations were found between age, family income, and sleep duration, while gender displayed a negative correlation with sleep efficiency, consistent with previous sleep pattern studies [36]. Importantly, CKD duration showed positive associations with sleep duration, sleep disturbance, anxiety, and depression levels, underscoring the long-term impact of CKD on mental health and sleep quality. Early intervention and comprehensive care are essential to mitigate these effects. Our findings stress the significance of considering demographic and clinical factors in developing targeted interventions for this patient group.

Our study found significant correlations and predictors for sleep quality, anxiety, depression, CKD duration, and HD duration among Somali HD patients. Longer HD treatment correlated with increased sleep latency and heightened depressive symptoms, emphasizing the need for ongoing mental health support for long-term HD patients. Moderate correlations were identified between various aspects of sleep quality, anxiety, and depression, indicating a bidirectional relationship between sleep problems and emotional well-being, consistent with prior research [37]. Anxiety and depression predicted subjective sleep quality, sleep latency, duration, and efficiency, underscoring the crucial role of addressing these psychological factors to improve sleep outcomes in HD patients. These findings highlight intricate relationships between sleep quality, anxiety, depression, and CKD and HD treatment factors, guiding targeted interventions for enhanced well-being in this patient population.

Our study identified robust predictors for sleep quality among HD patients, including CKD and HD duration, depression levels, family income, age, and gender. These factors have an enduring impact on sleep, necessitating continuous monitoring and tailored interventions, especially for patients with longer treatment histories. Socioeconomic factors, age, and gender play roles in sleep duration and efficiency, highlighting the need for targeted interventions. Additionally, anxiety predicted sleep medication usage, and both anxiety and depression predicted daytime dysfunction, emphasizing the impact of psychological distress on sleep-related behaviors and functioning.

The majority of our study participants were male, in line with previous research [38]. Additionally, nearly half of the participants were aged 55 or older, consistent with trends observed in similar studies [38]. This demographic pattern reflects the well-known association between older age and a higher prevalence of renal failure. One reason for this could be delayed diagnosis and treatment resulting from neglected chronic conditions like hypertension and diabetes, which, if untreated, can progress to renal failure over time. Our study confirmed this trend, with hypertension identified as the primary cause of CKD among our HD patients, closely followed by diabetes mellitus. These findings resonate with studies conducted in India and Ethiopia [1,39]. Our research delved into the complex relationship between CKD and HD duration and the psychological well-being of our patients. Unlike the findings reported by Gadia et al. [40], our study revealed a significant link between CKD duration and depression, echoing conclusions drawn by Roy et al. [41]. This connection underscores the enduring impact of CKD on the mental health of HD patients. As the disease progresses, individuals may experience heightened depressive symptoms due to various factors, including the burden of managing a chronic illness, fear of mortality, physical and social limitations, financial constraints, and dietary restrictions. Our study, consistent with prior research [38,40], established a significant link between prolonged HD treatment and increased depression and anxiety levels. Prolonged CKD and HD treatment bring persistent fear of mortality, additional physical and social limitations, financial pressures, and dietary restrictions. Coupled with potential severe side effects of long-term dialysis, these factors contribute to the emotional distress experienced by our patients. These findings emphasize the critical need for comprehensive care, addressing medical and psychological aspects, to support HD patients facing multifaceted challenges.

Our study revealed a notably high prevalence of depression among HD patients, with 61.5% scoring positive on the PHQ-9 and 37.5% displaying signs of depression based on the HADS-D. These rates are consistent with studies in Africa (44% to 72.5%) [7,9,10,39] and Asia (42.9% to 80.4%) [1,40,41,42], as well as reports from Netherlands, Nigeria, and Spain (34.8%, 35%, and 37%, respectively) [43,44,45]. This aligns with a systematic review’s depression prevalence range (22.8% to 39.3%) [46]. In contrast, our study’s anxiety prevalence was relatively lower than in Africa and Asia (40% to 61%) [7,40,47,48]. Variations in rates stem from factors like study populations, assessment methods, healthcare access, and cultural influences. Our findings differed from a narrative review, suggesting anxiety rates between 12% and 52% in HD patients [49], aligning instead with Zhang et al.’s report (10%) [50]. Surprisingly, our analysis found no significant gender-based differences in anxiety and depression scores among HD patients, echoing Aggarwal et al.’s findings [6]. This suggests that emotional challenges affect both genders equally. Lower anxiety and depression rates in our study might be due to Somali patients’ resilience and robust support networks. Cultural and social factors, including strong family and community ties, play pivotal roles in Somali patients’ emotional coping mechanisms. However, our results might be influenced by social desirability bias; patients might underreport symptoms due to stigma. These results emphasize the necessity of culturally sensitive mental health interventions for HD patients, addressing both clinical and emotional aspects of their well-being.

Our study found lower rates of poor sleep quality among HD patients compared to prior studies in African and Asian populations. Prevalence rates in our study differed from higher rates reported in previous African studies (ranging from 62.2% to 88%) [21,22,23] and Asian studies [16,51]. These variations stem from differences in patient characteristics, cultural influences, geographical factors, and variations in assessment tools used to evaluate sleep quality. The diverse nature of HD patient populations across regions contributes to variability in sleep quality prevalence rates. Demographic differences, underlying medical conditions, and sociocultural backgrounds can impact sleep disturbances. Additionally, the geographical and cultural context of HD treatment can influence patients’ sleep patterns and overall sleep quality. Variation in sleep assessment tools used in studies also contributes to reported prevalence differences. Sleep quality is multifaceted, and the choice of assessment tool affects measurement. Different tools capture different aspects of sleep disturbances, leading to variations in reported rates. Consistent with previous research [18,23], our study found no significant gender-based differences in sleep quality among HD patients, indicating consistent experiences of poor sleep quality between male and female patients. These findings align with prior studies exploring gender differences in sleep patterns among individuals with chronic illnesses.

Our study highlighted the profound impact of higher anxiety and depression levels on various aspects of the PSQI, with regression analyses confirming their significant influence on sleep quality in HD patients. Specifically, our findings indicated that depression, assessed through PHQ-9 and HADS-D, and anxiety, measured by HADS-A, were significant predictors of insomnia. Furthermore, depression remained a predictor of overall sleep quality even after considering age and gender. These results underscore the critical role played by depression and anxiety in disrupting the sleep patterns of HD patients, emphasizing the potential for enhanced sleep quality through the management of these psychological symptoms. Addressing anxiety and depression in the clinical care of HD patients could substantially improve their sleep quality and, consequently, their overall quality of life. In line with some previous research [4,21], our study established a direct correlation between sleep disturbance and both illness and HD duration. This finding suggests that the cumulative effect of living with a chronic illness over an extended period can contribute to sleep disturbances, particularly when coupled with the demands of ongoing HD treatment. Additionally, our study revealed significant correlations between depression, anxiety, poor sleep quality, and clinically significant insomnia, aligning with findings from prior research studies [4,6,21,52].

While the association between depression and poor sleep quality has consistent support in various studies, the link between anxiety and poor sleep quality is less uniform across the literature, warranting further investigation. Our cross-sectional study does not establish causation, and the relationships between anxiety, depression, and sleep quality may involve bidirectional influences and reverse causation. For example, depression and anxiety may contribute to poor sleep quality, but poor sleep quality can also exacerbate or trigger these psychological symptoms, creating a cyclical relationship. Our study provides valuable insights into the interplay between anxiety, depression, and sleep quality among HD patients, emphasizing the need for ongoing research to inform clinical care and interventions for this population.

Our study, while providing valuable insights into the relationships between anxiety, depression, and sleep quality among Somali HD patients, has several limitations that warrant consideration. First, it was conducted at a single HD center using a cross-sectional design and may not be representative of the general population. Similarly, the gender distribution in our study may not precisely mirror the general population of patients in Somalia. Second, sleep quality was evaluated using self-report scales rather than more objective measures such as polysomnography. Third, our study did not gather information on specific sleep complaints, such as restless leg syndrome, among HD patients. Lastly, we fully acknowledge that our study’s design inherently prevents us from establishing causality, and we want to emphasize that our findings represent associations rather than causal relationships. The interactions between depression, anxiety, and sleep quality are undeniably complex and bidirectional. While our research provides valuable insights into these relationships, we concur that future studies, including longitudinal investigations, are essential to further unravel the causative pathways at play.

## 5. Conclusions

Patients with kidney failure who are undergoing HD are particularly vulnerable to experiencing psychological difficulties. The findings of our study underscore the urgency of implementing routine psychological screening during HD visits for this vulnerable population. The early detection and treatment of anxiety, depression, and insomnia can improve their quality of life, enhance medication compliance, and reduce morbidity and mortality. Implementing standard screening practices is strongly recommended, to foster proactive intervention and comprehensive care. Collaboration among healthcare providers will help prevent psychological issues and promote well-being in this patient population. In sum, our study underscores the critical importance of routine psychological screening as a fundamental component of comprehensive care for kidney failure patients undergoing HD. Early detection and intervention not only improve psychological well-being but also contribute to better overall health outcomes, highlighting the need for proactive measures and collaboration among healthcare providers to support this vulnerable patient group.

## Figures and Tables

**Table 1:** Sociodemographic characteristics of the study participants (*n* = 200).

Variable	Category	*n*	%
Age (years)	18–24	9	4.5
	25–34	22	11.0
	35–44	28	14.0
	45–54	45	22.5
	55–64	69	34.0
	>65	27	13. 5
Gender	Female	83	41.5
	Male	117	58.5
Marital status	Single	12	6.0
	Married	139	69.5
	Divorced	24	12.0
	Widowed/Widower	25	12.5
Education status	Illiterate	137	68.5
	Intermediate	20	10.0
	Secondary	30	15.0
	University	13	6.5
Occupational status	Employed	188	94.0
	Unemployed	11	5.5
	Retired	1	0.5
Family income	Unknown	88	44.0
	1000–1500 dollars	22	11.0
	1500–2000 dollars	45	22.5
	>2000 dollars	45	22.5
Duration of CKD	<1 year	40	20.0
	1–3 years	69	34.5
	3–5 years	59	29.5
	>5 years	32	16.0
Cause of kidney failure	Hypertension Diabetes mellitus Glomerulonephritis Others	117	58.5
		40	20.0
		5	2.5
		38	19.0
Duration on HD	3 months	25	12.5
	1 year	46	23.0
	1–3 years	51	25.5
	3–5 years	55	27.1
	>5 years	23	11.5
Number of dialysis sessions per week	Once a week	21	10.5
	Twice a week	140	70.0
	Thrice a week	34	17.0
	Four times a week	5	2.5

CKD: chronic kidney disease.

**Table 2:** Descriptive characteristics for the PHQ-9, HADS, ISI, and PSQI dimensions (*n* = 200).

Variable	Median	IQR	Mean	SD	Minimum	Maximum	Cronbach’s Alphas
PHQ-9	6.00	4.00	6.01	3.39	0.00	17.00	0.76
HADS-A	2.00	4.00	2.79	3.07	0.00	17.00	0.84
HADS-D	6.00	6.00	6.07	3.19	0.00	15.00	0.75
ISI	1.00	12.00	5.81	7.65	0.00	25.00	0.74
PSQI Total	2.00	7.00	4.39	4.73	0.00	16.00	0.85
Quality of Sleep							Cronbach’s Alphas *
Subjective Sleep Quality	0.00	2.00	0.71	1.10	0.00	3.00	0.80
Sleep Latency	1.00	3.00	1.36	1.23	0.00	3.00	0.83
Sleep Duration	0.00	2.00	0.99	1.21	0.00	3.00	0.80
Sleep Efficiency	0.00	1.00	0.48	0.95	0.00	3.00	0.82
Sleep Disturbance	0.00	1.00	0.32	0.47	0.00	3.00	0.87
Sleep Medication	0.00	0.00	0.08	0.41	0.00	3.00	0.87
Daytime Dysfunction	0.00	1.00	0.44	0.75	0.00	3.00	0.81

IQR: interquartile range; SD: standard deviation; PHQ-9: Patient Health Questionnaire Version-9; HADS: HADS-A: Hospital Anxiety and Depression Scale-Anxiety subscale; HADS-D: Hospital Anxiety and Depression Scale-Depression subscale; ISI: Insomnia Severity Index; PSQI: Pittsburgh Sleep Quality Index; * Cronbach’s alpha coefficient of the scale when each item was deleted.

**Table 3:** Characteristics of participants according to depressive and anxiety symptoms, and sleep qualities (*n* = 200).

	No Anxiety/Depression		Mild Anxiety/Depression		Moderate Anxiety/Depression	Severe Anxiety/Depression
HADS-A	185 (92.5%)		9 (4.5%)		5 (3%)	1 (0.5%)
HADS-D	125 (62.5%)		62 (31%)		13 (6.5%)	0 (0%)
	No depression	Mild depression		Moderate depression	Moderate to severe depression	Severe depression
PHQ-9	77 (38.5%)	96 (48%)		23 (11.5%)	4 (2%)	0 (0%)
	Good sleepers				Poor sleepers	
PSQI	137 (68.5%)				63 (31.5%)	
	No insomnia		Sub-threshold insomnia		Moderate insomnia	Severe insomnia
ISI	141 (70.5%)		18 (9%)		30 (15%)	11 (5.5%)

HADS-A: Hospital Anxiety and Depression Scale-Anxiety subscale; HADS-D: Hospital Anxiety and Depression Scale-Depression subscale; PHQ-9: Patient Health Questionnaire Version-9; PSQI: Pittsburgh Sleep Quality Index; ISI: Insomnia Severity Index.

**Table 4:** Spearman’s rank-order test correlations (*r_s_*) between sleep quality measures, demographic characteristics, and psychometric scales.

	1	2	3	4	5	6	7	8	9	10	11	12	13
1. Insomnia Severity Index Scale Total Score	1.000												
2. Subjective Sleep Quality	0.815 **	1.000											
3. Sleep Latency	0.623 **	0.602 **	1.000										
4. Sleep Duration	0.805 **	0.754 **	0.609 **	1.000									
5. Sleep Efficiency	0.608 **	0.585 **	0.555 **	0.621 **	1.000								
6. Sleep Disturbance	0.339 **	0.352 **	0.327 **	0.295 **	0.190 **	1.000							
7. Sleep Medication Use	0.183 **	0.238 **	0.169 *	0.140 *	0.111	0.056	1.000						
8. Daytime Dysfunction	0.748 **	0.872 **	0.576 **	0.712 **	0.585 **	0.336 **	0.267 **	1.000					
9. PHQ-9 Total	0.611 **	0.633 **	0.453 **	0.572 **	0.402 **	0.287 **	0.124	0.596 **	1.000				
10. HADS–Anxiety	0.494 **	0.491 **	0.419 **	0.388 **	0.390 *	0.206 **	0.159	0.474 **	0.516 **	1.000			
11. HADS–Depression	0.586 **	0.529 **	0.502 **	0.533 **	0.419 **	0.393 **	0.038	0.554 **	0.716 **	0.606 **	1.000		
12. Duration of CKD	0.115	0.102	0.123	0.160 *	0.033	0.144 *	−0.064	0.136	0.221 **	0.262 **	0.351 **	1.000	
13. Duration of HD	0.095	0.072	0.148 **	0.138	0.036	0.030	−0.060	0.115	0.168 *	0.227	0.263 *	0.834 **	1.000

Note. * *p* < 0.05; ** *p* < 0.01; PHQ-9: Patient Health Questionnaire Version-9; HADS-A: Hospital Anxiety and Depression Scale-Anxiety subscale; HADS-D: Hospital Anxiety and Depression Scale-Depression subscale; CKD: chronic kidney disease; HD: hemodialysis.

**Table 5:** (**a**) Hierarchical linear regression for exploring the variables associated with sleep quality (ISI Scale). (**b**) Hierarchical linear regression for exploring the variables associated with sleep quality (PSQI Scale).

(a)											
	*F*	*Adjusted R*²	*Δ R*²	*B* (95% CI)	*SE*	*β*	*t*	*p*	*Durbin-* *Watson*	*Tolerance*	*VIF*
Step 1	1.011	0.000	0.025								
Age				0.557 (−0.339, 1.454)	0.455	0.088	1.226	0.222		0.965	1.036
Gender				−0.033 (−2.223, 2.158)	1.111	−0.002	−0.030	0.976		0.976	1.025
Income level				0.323 (−0.554, 1.200)	0.455	0.052	0.727	0.468		0.979	1.021
Duration of CKD				1.354 (−0.595, 3.302)	0.988	0.174	1.370	0.172		0.311	3.215
Duration of HD				−0.615 (−2.202, 0.972)	0.805	−0.098	−0.764	0.446		0.308	3.245
Step 2	60.348	0.380	0.402								
Age				0.508 (−0.207, 1.223)	0.362	0.081	1.401	0.163	1.844	0.942	1.061
Gender				0.391 (−1.336, 2.118)	0.876	0.025	0.446	0.656		0.973	1.027
Income level				0.093 (−0.599, 0.785)	0.351	0.015	0.264	0.792		0.975	1.026
Duration of CKD				−0.049 (−1.605, 1.506)	0.789	−0.006	−0.063	0.950		0.303	3.304
Duration of HD				−0.225 (−1.480, 1.029)	0.636	−0.036	−0.354	0.723		0.306	3.267
HADS anxiety				0.852 (0.517, 1.188)	0.170	0.342	5.014	<0.001		0.670	1.493
HADS depression				0.891 (0.560, 1.222)	0.168	0.372	5.307	<0.001		0.636	1.573
**(b)**											
	***F***	***Adjusted R*²**	***Δ R*²**	***B* (*95% CI*)**	***SE***	***β***	***t***	***p***	***Durbin-*** ***Watson***	***Tolerance***	***VIF***
Step 1	1.208	0.005	0.030								
Age				0.467 (−0.087, 1.020)	0.281	0.120	1.662	0.098		0.965	1.036
Gender				−0.189 (−1.542, 1.164)	0.686	−0.020	−0.275	0.784		0.976	1.025
Income level				0.136 (−0.405, 0.678)	0.275	0.035	0.497	0.620		0.979	1.021
Duration of CKD				0.400 (−0.803, 1.604)	0.610	0.083	0.656	0.512		0.311	3.215
Duration of HD				0.074 (−0.906, 1–055)	0.497	0.019	0.150	0.881		0.308	3.245
Step 2	64.794	0.400	0.391								
Age				0.412 (−0.023, 0.848)	0.221	0.106	1.868	0.063	1.745	0.942	1.061
Gender				−0.073 (−0.980, 1.125)	0.533	−0.008	−0.136	0.892		0.973	1.027
Income level				−0.013 (−0.435, 0.408)	0.214	−0.003	−0.062	0.950		0.975	1.026
Duration of CKD				−0.491 (−1.438, 0.457)	0.480	−0.102	−1.021	0.308		0.303	3.304
Duration of HD				0.304 (−0.460, 1.068)	0.387	0.078	0.785	0.434		0.306	3.267
HADS anxiety				0.474 (0.270, 0.678)	0.104	0.307	4.581	<0.001		0.670	1.493
HADS depression				0.622 (0.421, 0.824)	0.102	0.419	6.085	<0.001		0.636	1.573

CI: confidence interval; SE: standard error; VIF: variance inflation factor; ISI: Insomnia Severity Index scale; CKD: chronic kidney disease; HD: hemodialysis; HADS: Hospital Anxiety and Depression Scale; PSQI: Pittsburgh Sleep Quality Index scale.

## Data Availability

The datasets and materials in this study are available on request from the corresponding author.

## Abbreviations

CKD:	chronic kidney disease;
QoL:	quality of life;
HD:	hemodialysis;
RLS:	restless leg syndrome;
OSA:	obstructive sleep apnea;
PHQ-9:	Patient Health Questionnaire-9;
HADS:	Hospital Anxiety and Depression Scale;
ISI:	Insomnia Severity Index;
PSQI:	Pittsburgh Sleep Quality Index.
